# Angiotensin Receptor Antagonists to Prevent Sudden Death in Heart Failure: Does the Dose Matter?

**DOI:** 10.1155/2014/652421

**Published:** 2014-02-06

**Authors:** Pietro Francia, Francesca Palano, Giuliano Tocci, Carmen Adduci, Agnese Ricotta, Lorenzo Semprini, Massimo Caprinozzi, Cristina Balla, Massimo Volpe

**Affiliations:** ^1^Cardiology, Department of Clinical and Molecular Medicine, Sant'Andrea Hospital, Sapienza University, Via di Grottarossa 1035-39, 00189 Rome, Italy; ^2^I.R.C.C.S. Neuromed, Pozzilli, Italy

## Abstract

International guidelines recommend ICD implantation in patients with severe left ventricular dysfunction of any origin only after careful optimization of medical therapy. Indeed, major randomized clinical trials suggest that suboptimal use of fundamental drugs, such as ACE inhibitors (ACE-i) and beta-blockers, may affect ICD shock-free survival, sudden cardiac death (SCD), and overall mortality. While solid evidence in favour of pharmacological therapy based on ACE-i with or without beta-blockers is available, data on SCD in HF patients treated with angiotensin receptor blockers (ARBs) are limited. The present paper systematically analyses the impact of ARBs on SCD in HF and reviews the contributory role of the renin-angiotensin system (RAS) to the establishment of arrhythmic substrates. The following hypothesis is supported: (1) the RAS is a critical component of the electrical remodelling of the failing myocardium, (2) RAS blockade reduces the risk of SCD, and (3) ARBs represent a powerful tool to improve overall survival and possibly reduce the risk of SCD provided that high doses are employed to achieve optimal AT_1_-receptor blockade.

## 1. Introduction

Heart failure (HF) affects 15 million people in Europe, with a prevalence of 2-3% in the general population and 10–20% in 70- to 80-year-old subjects. It represents the common ending of different cardiovascular diseases and is characterized by high short-term mortality in advanced stages (up to 50% at 1 year for NYHA class IV patients) [1–4].

Death in HF occurs either from circulatory failure due to progressive left ventricular (LV) dysfunction or sudden cardiac death (SCD). This latter accounts for approximately half of all HF deaths, the underlying mechanism being sudden onset of ventricular tachycardia (VT) or ventricular fibrillation (VF). Despite decades of research for the evaluation of hundred compounds, there are no antiarrhythmic drugs that definitely prevent SCD in HF patients on already optimized therapy with *β*-blockers and ACE inhibitors [5]. In contrast, the implantable cardioverter-defibrillator (ICD) prevents SCD and improves survival in both primary and secondary prevention patients treated with optimal medical therapy, including beta-blockers and either ACE inhibitors or angiotensin receptor blockers (ARBs) [6–8].

Based on data from multiple randomized primary prevention trials, current guidelines recommend ICD implantation in patients with symptomatic and severe LV dysfunction of any origin on already optimized medical therapy [9]. However, the concept of optimal drug treatment in ICD trials has been questioned [10]. Indeed, most primary and secondary prevention studies show an increased risk of death or appropriate ICD interventions associated with an imbalanced use of antiarrhythmic drugs or *β*-blockers and ACE-inhibitors among the study arms [10]. Findings from these clinical trials suggested that suboptimal use of fundamental drugs, such as ACE inhibitors and beta-blockers, may impact on event-free survival, SCD, and overall mortality. With the exception of the SCD-HeFT [8], DINAMIT [11], DEFINITE [12], and MADIT II [7] trials, only 8 to 50% of patients enrolled in major primary prevention studies were on *β*-blockers, and less than 70% of patients were treated with ACE-i. While solid evidence in favour of pharmacological therapy based on ACE-i with or without beta-blockers is available, data on SCD in HF patients treated with ARBs are limited.

The present paper systematically analyses the impact of ARBs on SCD in HF and reviews the contributory role of the renin-angiotensin system (RAS) to the establishment of proarrhythmic substrates.

## 2. ARBs and Sudden Death in Clinical Trials

The ELITE study [13] was originally designed to compare the effects of losartan (50 mg/day) and captopril (50 mg three times daily) on renal function in HF patients. Although renal outcomes were similar in the two groups, the results of a secondary endpoint analysis showed a 45% reduction in total mortality in HF patients randomized to losartan compared to placebo, mainly driven by a 36% decrease in SCD [13]. As the superior effects of losartan were based on a small number of events that were not the primary endpoint, a larger randomised trial, the ELITE II [14] (Table 1), was specifically designed to evaluate mortality. In this trial, losartan 50 mg once daily did not prove superior efficacy as compared to captopril 50 mg three times daily and showed a trend towards higher incidence of sudden death or resuscitated cardiac arrest (HR: 1.25; 95% CI: 0.98–1.60; *P* = 0.08) [14].

The Val-HeFT trial [15] (Table 1) was a randomized, placebo-controlled, double-blind, and parallel-group trial, evaluating the long-term effects of the addition of valsartan to standard therapy in more than 5.000 patients with HF. Eligible patients included in this trial had to have been receiving for at least two weeks a fixed-dose drug regimen that could include ACE inhibitors, diuretics, digoxin, and beta-blockers. The primary outcomes were mortality and the combined endpoint of mortality and morbidity, defined as the incidence of cardiac arrest with resuscitation, hospitalization for heart failure, or receipt of intravenous inotropic or vasodilator therapy. Although overall mortality was similar in the two groups, valsartan reduced the risk of first hospitalisation for HF by 34.4% (*P* = 0.0007) as compared to placebo. In addition, resuscitation of cardiac arrest was improved with valsartan, without achieving statistically significance (0.6 versus 1.0%, *P* = ns). However, at the time of randomization, about 93% of patients were on ACE inhibitors in both treatment arms, not allowing to assess the isolated benefits of ARBs.

In the OPTIMAAL trial [16] (Table 1), comparing captopril and losartan in high-risk patients after acute myocardial infarction, all-cause mortality was nonstatistically different in the two study arms and showed a trend towards higher incidence of death (RR 1.13; 95% CI 0.99–1.28; *P* = 0.07) and SCD (RR 1.19; 95% CI 0.98–1.43; *P* = 0.07) in the losartan as compared to captopril group.

The VALIANT study [17] (Table 1) randomized 14.703 patients with myocardial infarction complicated by HF, left ventricular dysfunction or both to valsartan 160 mg twice daily, captopril 50 mg three times daily, or captopril 50 mg three times daily plus valsartan 80 mg twice daily. The primary endpoint of the study was death from any cause. The results showed noninferiority of valsartan compared with that of captopril. In a post hoc analysis of the risk and time course of SD in the VALIANT study population [18], 1067 patients (7%) experienced SD (*n* = 903) or resuscitated cardiac arrest (*n* = 164) in a median of 180 days after MI. The risk was the highest in the first 30 days after MI (1.4% per month) and was decreased (0.14% per month) after 2 years. Unfortunately, this analysis did not address which of the drug regimens was more effective in preventing SD or resuscitated cardiac arrest.

The CHARM programme [19], including three trials, reported that ARBs provide incremental benefit over background therapy with ACE-i in HF [20] and improve major endpoints when used as a replacement therapy in patients intolerant to ACE-i [21]. Indeed, in subjects who are intolerant to ACE-i, the CHARM Alternative trial [21], demonstrated that candesartan reduces cardiovascular death and hospital admissions for congestive HF as compared to the placebo group. Moreover, a post hoc analysis evaluating cause-specific mortality [22] (Table 1) revealed that the major reduction in cardiovascular mortality observed in the candesartan arm was ascribed to fewer SCD and HF deaths, accounting for 35% and 26% of all deaths, respectively.

The low doses of ARBs used in the former trials may at least in part explain the higher mortality as compared to that observed in the CHARM overall programme. This hypothesis is also supported by the recent findings of a registry-based cohort study [23] assessing whether losartan was associated with increased all-cause mortality in HF patients as compared with candesartan. Overall, drug therapy with losartan was not associated with higher mortality. However, mortality was higher when losartan was administered at low doses [23].

All these studies suggest that undertitration of ARBs may be associated with a poor outcome. Less information is given if beta-blocker cotreatment influences the dose response of ARBs. Of note, patients enrolled in all the above-mentioned trials were on optimized medical treatment including beta-blockers, the only drugs that proved to be effective in reducing sudden cardiac death, cardiovascular death and all cause mortality in HF [24]. In VALIANT, OPTIMAAL and COMPANION trials, the percentage of patients on beta-blockers was indeed 70% on average. To date, there are no published studies addressing the specific question whether beta-blockers modify the risk of sudden death in patients treated with suboptimal doses of ARBs.

## 3. ARBs in ICD Recipients with HF

Among clinical trials that demonstrated a significant survival benefit of the ICD [6, 8, 25], only SCD-HeFT reported on the use of ARBs (15% of patients at study entry) [8]. However, there are no available follow-up data of this study subgroup.

In a post hoc analysis of the COMPANION trial (Table 1), Saxon et al. [26] reported that both ACE-i and ARBs lowered the risk of appropriate ICD therapy. However, they did not report whether patients on ARBs were also on an ACE-i or whether ARBs were used at high or low doses. Providing this information is crucial when interpreting results.

In a population of ICD recipients with left ventricular ejection fraction <35% and no prior documented ventricular arrhythmias, Obeyesekere et al. [27] (Table 1) reported that appropriate device therapy occurred more frequently in patients without background therapy with ACE-i or ARBs. We recently conducted a prospective study to identify determinants of appropriate ICD interventions in a cohort of patients with HF implanted with a primary prevention ICD (Table 1). Interestingly, we found that patients treated with low-dose ARBs (75% assuming losartan at a mean dose of 51 mg/day) had a 2.4-fold increased risk of appropriate ICD intervention. This emphasizes that ARBs uptitration plays a key role in the protection against ventricular arrhythmias and possibly SCD [28].

## 4. Magnitude of RAS Blockade

Based on the pharmacodynamic profile of ARBs, dosages required to achieve a complete blockade of the AT_1_-subtype receptor (e.g., lack of vasoconstrictor response to a challenge with angiotensin II) are higher than those commonly used in clinical practice. In their experience on normotensive healthy volunteers, Mazzolai et al. [29] found that oral administration of losartan 50 mg provides only 35 to 45% blockade of AT_1_ receptors. This is consistent with findings from Maillard et al. [30], who reported on the effects of losartan, valsartan, and candesartan dosing on AT_1_ receptor blockade. In their study, oral administration of losartan 50 mg induced only 50% AT_1_ receptor blockade, which was comparable to valsartan 80 mg. Blockade of 67% of AT_1_ receptors required valsartan 160 mg.

Notably, main trials in HF patients allowed ARB doses that are considerably higher than those traditionally employed in clinical practice. In the Val-HeFT [15] the target daily dose of valsartan was 320 mg, and the CHARM studies [19–21, 31] allowed candesartan at a target dose of 32 mg/day. With specific regard to losartan, the HEAAL study [32] (Table 1) assessed whether 150 mg/day was superior to 50 mg/day in reducing morbidity and mortality in a cohort of 3.846 symptomatic HF patients. After a median follow-up of 4.7 years, losartan 150 mg/day significantly reduced hospital admissions for HF (HR 0.87; 95% CI: 0.76–0.98; *P* = 0.025) and showed a nonsignificant trend towards reduction in mortality (HR 0.94; 95% CI: 0.84–1.04; *P* = 0.24) as compared with losartan 50 mg/day. Among components of primary endpoint, SCD and progressive HF represented the most common causes of death (37% and 24% of deaths, resp.).

## 5. Electrical Remodelling and RAS Blockade: Focus on Mechanisms

**Figure 1:**
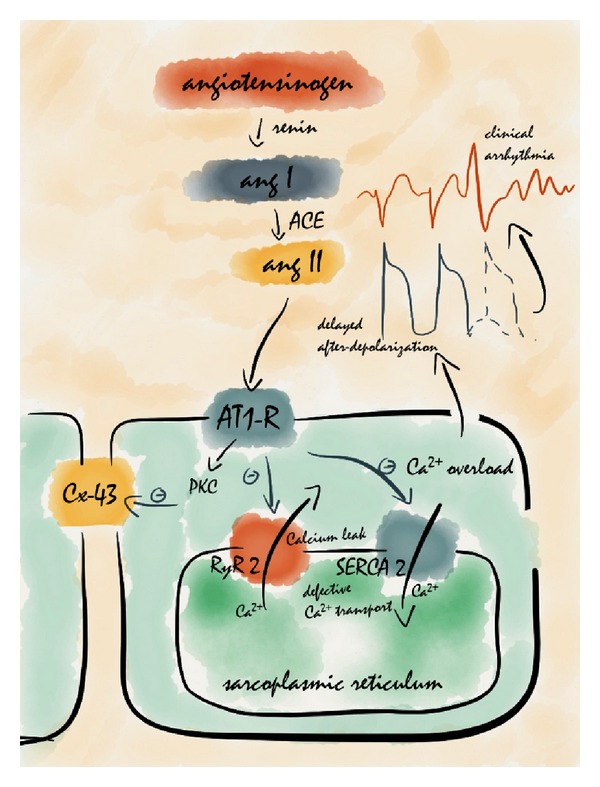
Molecular mechanisms that link AT_1_ receptor and arrhythmia susceptibility are summarized on this picture. The AT_1_ receptor downregulates connexin 43 via protein kinase C (PKC), thus favouring fragmentation and electrical reentry of the stimuli that can lead to tachyarrhythmias. Angiotensin II also affects Ca^2+^ homeostasis *via* the AT_1_ receptor by downregulating sarcoplasmic reticulum (SR) Ca^2+^-ATPase pump (SERCA 2a) and altering ryanodine receptor 2 (RyR2) function. Downregulation of SERCA2a through the AT_1_ receptor determines defective SR Ca^2+^ reuptake and promotes protein kinase A (PKA) phosphorylation of RyR2, thus leading to diastolic Ca^2+^ leak from the SR. The resultant cytoplasmic Ca^2+^ overload can trigger spontaneous delayed afterdepolarizations and ventricular arrhythmias.

Myocardial remodelling of the failing heart involves progressive loss of tissue due to necrosis and fibrosis, leading to inhomogeneous electrical conduction and offering the ideal *milieu* for triggering and propagation of ventricular arrhythmias. Local RAS expression contributes substantially to myocardial structural changes that favour cardiac arrhythmias [33]. Angiotensin II exerts its arrhythmogenic effects via multiple mechanisms that may involve or not the AT_1_ receptor. Among AT_1_ receptor-mediated mechanisms, angiotensin II decreases gap junction conductance *via* protein kinase C activation [34], shortens the refractory period by reducing the action potential duration [35], and modifies calcium conductance in cardiomyocytes [36]. Indeed, angiotensin II affects intracellular calcium homeostasis by targeting sarcoplasmic reticulum Ca^2+^-ATPase pump and ryanodine receptor [37, 38], thus promoting sarcoplasmic reticulum (SR) Ca^2+^ leak and defective SR Ca^2+^ reuptake during diastole. The resultant cytoplasmic Ca^2+^ overload can trigger delayed afterdepolarizations and ventricular arrhythmias [39, 40] (Figure 1). As AT_1_ receptors are widely expressed in the cardiac conduction system, angiotensin II may promote electrical instability also via enhanced spontaneous activity trough activation of the AT_1_ receptor in the sinoatrial/atrioventricular node and Purkinje fibres. Indeed, in isolated cardiac Purkinje fibres, angiotensin II increases the height and duration of the plateau phase of the action potential and promotes an inward shift in membrane current [41].

The increase in sympathetic activity via catecholamine release is among the main non-AT_1_ receptor-mediated arrhythmogenic effects of angiotensin II. Indeed, angiotensin II favours sympathetic neurotransmission in cardiac nerve terminals via inhibition of norepinephrine reuptake of neuronal cells [42] and suppression of vagal discharge of carotid sinus baroreceptor fibres [43]. Additional secondary arrhythmogenic mechanisms may involve direct tissue damage. As reported [44], infusion of angiotensin II in rabbits produces focal areas of myocardial necrosis probably due to the high sensitivity of coronary arteries to the vasoconstrictor effect of angiotensin II. Accordingly, necrosis/scarring of the conduction system may result, and ultimately lead to cardiac arrhythmias.

Dysfunctioning gap junctions are also likely to be involved in arrhythmogenis driven by angiotensin II. In an animal model of myocardial infarction, Kieken et al. [45] showed that angiotensin II upregulates c-Src tyrosine kinase, leading to downregulation of connexin 43 (Cx43), a major component of gap junction architecture. Loss of Cx43 causes fragmentation and slowdown of electrical stimuli, thereby favouring electrical reentry and tachyarrhythmias. Recently, Sovari and colleagues [46] demonstrated that, in a transgenic mouse model overexpressing cardiac angiotensin-converting enzyme (ACE 8/8 mice), c-Src tyrosine kinase inhibition prevents Cx43 loss, thus reducing susceptibility to cardiac arrhythmia. Interestingly, RAS blockade with losartan and captopril in ACE 8/8 mice induced a 2.4- and 2.3-fold increase in total cardiac Cx43 expression [47].

It is noteworthy that the contributory role of AT_2_ receptors in establishing life-threatening arrhythmic substrates or providing antiarrhythmic effects is poorly addressed at present. In animal models, AT_1_ receptor blockade with losartan significantly increases AT_2_ receptor mRNA [48]. Moreover, when experimental myocardial infarction is induced, the AT_2_ receptor agonist compound 21 (C21) reduces scar size by diminishing Fas-ligand and caspase-3 expression in the peri-infarct zone, indicating an antiapoptotic effect [49]. It seems reasonable that reducing scar size may also entail antiarrhythmic effects.

Both ACE-i and ARBs counteract the proarrhythmic effects of angiotensin II. Indeed, ACE-i prevent the conversion of angiotensin I to angiotensin II, while ARBs directly block AT_1_ receptors. As compared to ACE-i, ARBs have the advantage to inhibit the production of angiotensin II via ACE-independent pathways, providing a long-lasting effect that overwhelms possible loss of efficacy of ACE-i after prolonged use. Furthermore, ACE-i may increase bradykinin levels, which in turn trigger norepinephrine release leading to higher susceptibility to cardiac arrhythmias. Conversely, ARBs do not hydrolize ACE, thus preventing bradykinin increase.

## 6. Conclusions

The RAS contributes substantially to myocardial electrical remodelling of the failing heart, favouring ventricular arrhythmias. In experimental settings, RAS blockade through ACE-i and ARBs [39, 47, 50] displays antiarrhythmic properties that are both dependent on and beyond AT_1_ receptor pathways. In the clinical arena, drug treatment with ARBs represents a powerful tool to reduce the risk of cardiac arrhythmias, provided that full drug doses are employed. Indeed, randomized controlled trials showed that high doses of ARBs (valsartan 160–320 mg and candesartan 16–32 mg) are effective in improving survival in HF and may be therefore warranted also to reduce the risk of SD. On the contrary, using ARBs in this setting at suboptimal doses provides less than optimal receptor blockade, which turns to be clinically inadequate in severely diseased patients. In this view, the efficacy of high-dose ARBs in protecting HF patients from life-threatening arrhythmias should be prospectively assessed.

## Figures and Tables

**Table 1:** Clinical studies assessing the impact of ARBs on SCD, RCA,or appropriate ICD intervention.

Study	Aim of the study or primary endpoint	SCD	Mean daily dose of ARB	Results	
ELITE II [14]	Losartan versus captopril to improve survival in patients with NYHA II–IV and FE ≤ 40%	Secondary endpoint	Losartan 50 mg versus captopril 50 mg t.i.d.	Losartan not superior for mortality Higher incidence of SCD or RCA with losartan	HR 1.25 CI 95% (0.98–1.60) *P* = 0.08

Val-HeFT [15]	Valsartan for mortality and morbidity in NYHA II–IV		Valsartan 160 mg b.i.d. versus placebo	Total mortality similar in the two groups RCA improved with valsartan	RR 1.02 CI 97.5% (0.88–1.18) *P* = 0.80 0.6% versus 1.0%

OPTIMAAL [16]	Losartan versus captopril to decrease all-cause mortality after acute MI	Secondary endpoint	Losartan 50 mg versus captopril 50 mg t.i.d.	Trend in favor of captopril (death from any cause) Higher incidence of SCD or RCA with losartan	RR 1.13 CI 95% (0.99–1.28) *P* = 0.07 RR 1.19; CI 95% 0*·*98–1*·*43; *P* = 0.07

VALIANT [17]	Valsartan versus captopril in patients with MI associated with HF and/or LVD		Valsartan 160 mg b.i.d. versus captopril 50 mg t.i.d. versus valsartan + captopril	Valsartan noninferior to captopril for total mortality	HR 1.00 CI 97.5% (0.90–1.1) *P* = 0.98

CHARM post hoc analysis [22]	Candesartan for cause-specific mortality in HF patients	—	Candesartan titrated to 32 mg versus placebo	Reduction of SCD with candesartan	HR 0.85 CI 95% (0.73–0.99) *P* = 0.036

HEAAL [32]	Losartan 50 mg versus 150 mg for death or admission for HF	—	Losartan 50 mg versus losartan 150 mg	Reduction of death or admission for HF with 150 mg No effects on mortality	HR 0.90, 95% CI 0.82–0.99; *P* = 0.02 HR 0.94, 95% CI 0.84–1.04; *P* = 0.24

COMPANION post-hoc analysis [26]	Predictors of SCD or ICD intervention in patients receiving CRT	—	Unknown	Both ACE-i and ARBs reduced the risk of appropriate shocks	ACE-i: HR 0.44 CI 95% (0.26–0.75) *P* < 0.01 ARBs: HR 0.53 CI 95% (0.28–0.996) *P* 0.05

Obeyesekere et al. [27]	Predictors of appropriate ICD interventions in a primary prevention population	—	Unknown	Absence of ACE-i/ARBs predicts appropriate ICD intervention	OR 0.06 CI 95% (0.01–0.37) *P* < 0.003

Francia et al. [28]	Predictors of appropriate ICD interventions in a primary prevention population	—	Losartan 50 mg (75% of patients)	Low-dose ARBs associated with higher risk of ICD intervention	HR 2.9 CI 95% (1.1–7) *P* = 0.02

ARBs: angiotensin receptor antagonists; SCD: sudden cardiac death; RCA: resuscitated cardiac arrest; CRT: cardiac resynchronization therapy.
